# Glutamate acts as a neurotransmitter for gastrin releasing peptide-sensitive and insensitive itch-related synaptic transmission in mammalian spinal cord

**DOI:** 10.1186/1744-8069-7-47

**Published:** 2011-06-24

**Authors:** Kohei Koga, Tao Chen, Xiang-Yao Li, Giannina Descalzi, Jennifer Ling, Jianguo Gu, Min Zhuo

**Affiliations:** 1 Department of Physiology, Faculty of Medicine, University of Toronto, 1 King's College Circle, Toronto, Ontario M5S 1A8, Canada; 2 Center for Neuron and Disease, Frontier Institute of Science and Technology, Xi'an Jiaotong University, 28 Xianning West Road, Xian, Shaanxi 710049, China; 3 Department of Anesthesiology, University of Cincinnati College of Medicine, 231 Albert Sabin Way, Cincinnati, Ohio 45267-0531, USA

## Abstract

Itch sensation is one of the major sensory experiences of human and animals. Recent studies have proposed that gastrin releasing peptide (GRP) is a key neurotransmitter for itch in spinal cord. However, no direct evidence is available to indicate that GRP actually mediate responses between primary afferent fibers and dorsal horn neurons. Here we performed integrative neurobiological experiments to test this question. We found that a small population of rat dorsal horn neurons responded to GRP application with increases in calcium signaling. Whole-cell patch-clamp recordings revealed that a part of superficial dorsal horn neurons responded to GRP application with the increase of action potential firing in adult rats and mice, and these dorsal horn neurons received exclusively primary afferent C-fiber inputs. On the other hands, few A_δ _inputs receiving cells were found to be GRP positive. Finally, we found that evoked sensory responses between primary afferent C fibers and GRP positive superficial dorsal horn neurons are mediated by glutamate but not GRP. CNQX, a blocker of AMPA and kainate (KA) receptors, completely inhibited evoked EPSCs, including in those Fos-GFP positive dorsal horn cells activated by itching. Our findings provide the direct evidence that glutamate is the principal excitatory transmitter between C fibers and GRP positive dorsal horn neurons. Our results will help to understand the neuronal mechanism of itch and aid future treatment for patients with pruritic disease.

## Background

The spinal cord plays important roles in pain [1,2] as well as itch [3,4]. Itch sensation is conveyed to superficial spinal dorsal horn via C fibers [3,5]. Recently, gastrin releasing peptide (GRP) has been proposed to be a key neurotransmitter for itch sensation using molecular and behavioral approaches [6,7]. This hypothesis is mainly based on the evidence that GRP receptors (GRPRs) are expressed in the superficial dorsal horn, and pharmacological inhibition and genetic deletion of GRP signaling pathways reduce behavioral itch-like responses [6,7].

The hypothesis of GRP as a selective itch neurotransmitter is questioned by several recent studies using gene manipulated mice lacking vesicular glutamate transporter subtype 2 (VGLUT2) or *BHLHB5 *mutant mice. In the study of *BHLHB5 *mutant mice, loss of inhibitory interneurons in the spinal dorsal horn contributed to elevated itch responses [8], indicating that enhanced glutamate transmission within the circuit due to the dis-inhibition may contribute to abnormal itching. Conditional knockout of VGLUT2 in the dorsal root ganglion (DRG) neurons reduced neuronal responses of dorsal horn neurons to noxious stimulation [9]. While in mice with specific deletion of VGLUT2 in Nav1.8- or vanilloid receptor-positive DRG cells, behavioral responses to itch stimuli were surprisingly enhanced [10,11]. These findings suggest that the reduction or abolishment of glutamate release in the subpopulation of dorsal horn synapses may enhance itch responses, indicating that the fine regulation of glutamate release from primary afferent fibers, such as C and/or A_δ _fibers may be critical for the transmission of itch information in the spinal cord. However, the direct relationship between GRP and glutamate mediated excitatory synaptic transmission at the dorsal horn has not been reported. Whether or not GRP and/or glutamate serve as a transmitter for itch sensation in the spinal cord is thus questioned.

In this study, we used integrative electrophysiological approaches to investigate the potential itching transmitter in the spinal dorsal horn. First, we observed the sensitivity of GRP in the superficial dorsal horn neurons of rats and mice. Next, we identified whether the GRP sensitive or insensitive neurons were receiving monosynaptic C or A_δ _projections, and detected if the primary afferents evoked responses were glutamatergic transmission. Furthermore, transgenic mice was used in which the expression of green fluorescent protein (GFP) is controlled by the promoter of the *c-fos *gene, so that we recorded intradermal itch-stimulation activated FosGFP positive neurons in the dorsal horn, and identified that the glutamate is the major excitatory transmitter for the itch activated neurons. Our results provide the first evidence of glutamate-mediated excitatory transmission between sensory unmyelinated C fibers and GRP responsive neurons in the superficial dorsal horn.

## Results

### GRP sensitive dorsal horn neurons acutely dissociated from rat spinal cord

**Figure 1:**
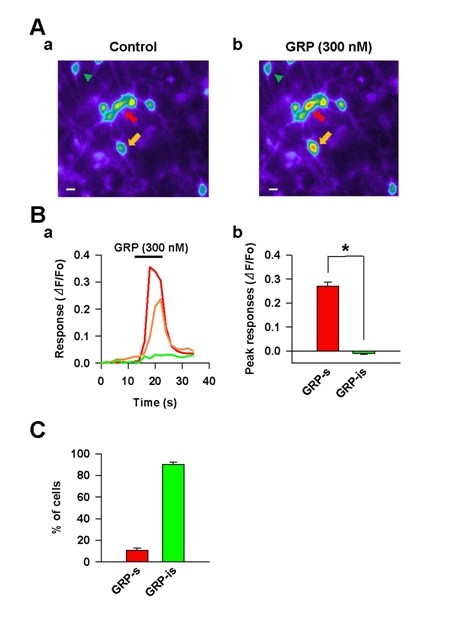
**GRP evoked responses in cultured dorsal horn neurons**. (A) Two sample images show a subpopulation of dorsal horn neurons responded to GRP. Control (before GRP application) is shown in (a) and the response to 300 nM GRP is shown in (b). Two arrows (red and orange) indicate two GRP-sensitive neurons (GRP-s), and an arrowhead (green) indicates a GRP-insensitive neuron (GRP-is). (B) GRP responses expressed as changes of Fluo-3 intensity (ΔF/F_0_) in the three cells shown in (a). Pooled results of peak Fluo-3 intensity (ΔF/F_0_) in GRP-s (red and orange line) and GRP-is (green line) neurons following the application of GRP (10 sec) in (b). Relative fluorescence intensity (ΔF/F_0_) was used to represent GRP responses and neurons with ΔF/F_0 _values of ≥ 0.15 (i.e., equal or above 15% baseline fluorescence intensity) were considered as responsive cells [12]. * *P *< 0.01, significant difference between GRP-s and GRP-is. (C) Percent of cells those are sensitive and insensitive to GRP.

We first performed calcium (Ca^2+^) imaging studies to map the possible GRP sensitive neurons in the rat dorsal horn (n = 9 tests, Figure 1). We prepared spinal culture neurons and applied a GRP receptor agonist (GRP, 300 nM, for 10 sec) in the culture (Figure 1A). Neurons with ΔF/F_0 _values of ≥ 0.15 (i.e., equal or above 15% baseline fluorescence intensity) were considered as responsive cells [12]. The application of GRP resulted in an increase of intracellular Ca^2+ ^concentration in a subpopulation of dorsal horn neurons as manifested by an increase of the Fluo-3 fluorescent intensity (GRP sensitive: 0.270 ± 0.018, GRP insensitive: -0.010 ± 0.003, unpaired *t *test, *P < 0.01, Figure 1A&1B). In nine tests on a total of 613 cells, 9% (8.8 ± 2.2%, n = 54/613 cells) of cells were shown to be GRP-sensitive (Figure 1C). The remaining cells showed no significant change in Fluo-3 intensity and were thereby considered to be GRP-insensitive (91.2 ± 2.2%, n = 5, 59/613 cells, Figure 1C).

### GRP elicited action potentials in superficial dorsal horn neurons of rats and mice

**Figure 2:**
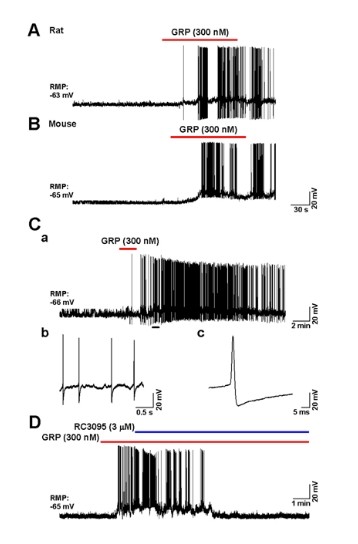
**GRP elicited firing in superficial dorsal horn**. Bath applications of GRP (300 nM for 2 min) elicited action potentials (APs) on parts of the neurons in rats (A) and mice (B). (C) An example of a bath applied GRP (300 nM for 2 min) produced long-lasting APs in superficial dorsal horn of a rat. The expanded APs in the long-lasting effect (b & c). (D) A GRP antagonist (RC3095, 3 μM) blocked GRP-induced APs.

GRP is expressed in DRG neurons, and the expression of GRPRs has been confirmed in the superficial dorsal horn, where GRP positive fibers are restricted to laminae I and II [6,7]. In order to identify the function of GRP in superficial dorsal horn neurons, we performed whole-cell patch-clamp recordings using spinal cord slice preparations in rats (n = 34) and mice (n = 9). If the recorded neurons produced action potentials (APs) from their resting membrane potentials by bath applications of GRP, we regarded as GRP-positive (sensitive) neurons. We found that bath applications of GRP (300 nM for 2 min) increased APs firing of neurons from both rats (n = 17/64, 26.6%) and mice (n = 6/23, 26.1%) (Figure 2A&2B). Furthermore, in some neurons, GRP induced long-lasting APs firing for more than 30 min in dorsal horn neurons of rats (36.8 ± 5.9 min, maximum: 57 min, n = 5/17 and mice (37.5 ± 12.5 min, maximum: 50 min, n = 2/6) (see Figure 2C, for an example). To confirm the contribution of GRP to the increasing of APs firing, we further used RC3095 (3 μM), a GRPR antagonist to block the activities of GRPRs, we found that bath applications of RC3095 blocked GRP induced APs firing (n = 4, Figure 2D).

The other dorsal horn neurons did not show any significant changes (rat: n = 47/64, 73.4%; mouse: n = 17/23, 73.9%) during GRP application, indicating that GRP selectively activates a subset of dorsal horn neurons. The difference in the percentages of GRP responsive cells in culture vs. *in vitro *spinal cord slices may be due to the different pools of cell recorded. In the case of dorsal horn cultures, all dorsal horn neurons were included, while in spinal cord slices, electrophysiological recordings were only performed from the superficial dorsal horn lamina.

### GRP sensitive dorsal horn neurons receiving monosynaptic C fiber input

**Figure 3:**
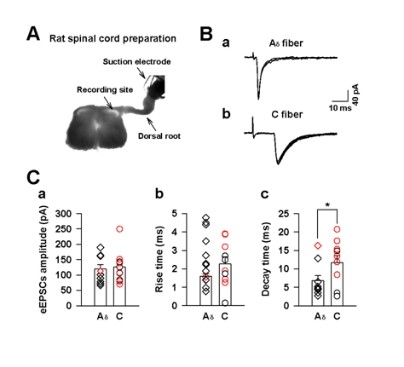
**GRP sensitive spinal dorsal horn neurons mostly receives sensory inputs from C fibers**. (A) Digitized photomicrograph showing one example for whole cell patch recording on neurons in the superficial lamina of spinal cord, which was stimulated by dorsal roots. (B) Examples of A_δ _(a) and C fiber (b) evoked monosynaptic EPSCs. (C) The amplitude (a), rise time (b) and decay time (c) of A_δ _and C fiber evoked EPSCs. The Red and black showed GRP sensitive and insensitive neurons, respectively. * *P *< 0.05, significant difference between A_δ _and C fiber.

Itch sensation is believed to be conveyed to the superficial dorsal horn via unmyelinated C fibers [3,13,14]. GRP is expressed in a subset of small and medium-sized DRG neurons [6]. To determine the sensory inputs for GRP responsive dorsal horn neurons, we next prepared lumbar spinal slices attached with dorsal root in rats (Figure 3A). First, we examined if the recorded superficial neurons received monosynaptic A_δ _and/or C afferent inputs in voltage-clamp mode (holding at -60 mV) (Figure 3B). The A_δ _or C fiber-evoked EPSCs were distinguished on the basis of the conduction velocity of afferent fibers (A_δ_, 2-13 m/s; C, < 0.8 m/s), and monosynaptic responses were identified by measuring no failure by repetitive dorsal root stimulations (20 Hz, 20 times for A_δ _and 2 Hz, 20 times for C fiber) [15]. After identifying responses to the stimulation of A_δ _and/or C afferent inputs, we applied GRP to the neurons in current-clamp mode (I = 0). For neurons receiving monosynaptic A_δ _fibers inputs, we found that only one in ten neurons was activated by GRP (n = 1/10, 10%, Figure 3C). In contrast, for neurons receiving monosynaptic unmyelinated C fiber inputs, six in ten cells were activated by GRP (n = 6/10, 60%, Figure 3C). The amplitude and rise time of evoked EPSCs induced by stimulating primary afferent A_δ _fibers (amplitude: 119.7 ± 14.4 pA, rise time: 1.6 ± 0.2 ms, n = 10) were not significantly different from that induced by primary afferent C fibers (amplitude: 125.6 ± 16.4 pA, rise time: 2.3 ± 0.4 ms, n = 10, Figure 3Ca&3Cb). On the other hand, the decay time constants in A_δ _fibers (decay time: 6.8 ± 1.4 ms, n = 10) was significantly different from that induced by primary afferent C fibers (decay time: 11.7 ± 1.9 ms, n = 10, unpaired *t *test, *P < 0.05, Figure 3Cc). Furthermore, in neurons receiving monosynaptic C fibers, the decay time of GRP sensitive neurons had significantly slower kinetics compared to that of GRP insensitive neurons (decay time in GRP sensitive neurons: 14.6 ± 1.8 ms, n = 6; in GRP insensitive neurons: 7.3 ± 2.8 ms, n = 4, unpaired *t *test, *P < 0.01).

### Glutamate is the transmitter for GRP sensitive dorsal horn neurons

**Figure 4:**
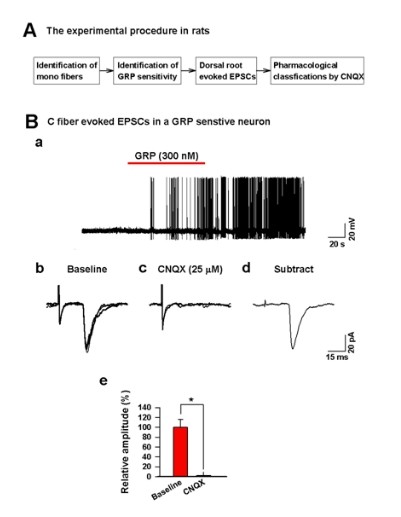
**C fiber evoked responses in GRP positive neurons are blocked by glutamate antagonists**. (A) The experimental procedure to identify the transmitter between C fibers and GRP positive neurons in rats. (B) In a GRP sensitive neuron (a), a monosynaptic C fiber-evoked EPSCs (b) was totally blocked by a bath application of CNQX (25 μM), a AMPA/KA receptor antagonist (c-e, n = 4).

Based on the analyses of current kinetics (see above), we found that the kinetics of EPSCs recorded from GRP responsive dorsal horn neurons are similar to those of AMPA/KA receptor mediated EPSCs in dorsal horn [16]. To test if the evoked responses are mediated by glutamate receptors, we performed electrophysiological experiments using a selective pharmacological antagonist. As shown in Figure 4A, we first identified monosynaptic responses of dorsal horn neurons to the stimulation of the dorsal root in voltage-clamp mode. After characterizing monosynaptic responses, we then applied GRP under current-clamp mode to detect any neuronal responses. In 64 dorsal horn neurons recorded of rats, we found that 17 cells were GRP sensitive (Figure 4Ba). Among these cells, we then recorded the dorsal root evoked EPSCs in voltage-clamp mode. We found that unmyelinated monosynaptic C fiber evoked EPSCs in the GRP sensitive neurons were completely blocked by CNQX (25 μM), a glutamatergic AMPA/KA receptor antagonist (100 ± 16.4% in baseline; 1.5 ± 0.8% in CNQX, unpaired *t *test, *P < 0.05, n = 4, Figure 4Bb-e). These results suggest that glutamate is the excitatory transmitter which mediates the synaptic transmission between afferent C fibers and GRP sensitive neurons in the spinal cord.

### GRP does not significantly contribute to summated EPSCs

**Figure 5:**
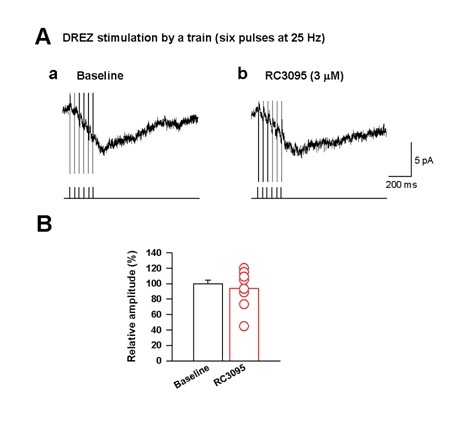
**GRP does not contribute to the summation of excitatory postsynaptic currents (EPSCs)**. (A) (a) Examples of synaptic responses induced by one train of stimulation containing six pulses delivered at 25 Hz (18 V) at DREZ in the presence of CNQX (25 μM), AP-5 (50 μM), Picrotoxin (100 μM) and Strychine (2 μM). A bath application of a GRPR antagonist (RC3095, 3 μM) was ineffective to the summated EPSCs (Ab). Bottom bars in (a&b) showing stimulation timing. (B) Summary data for A (n = 8, respectively). Red circles show each value.

The release of neuropeptides such as substance P in the spinal cord dorsal horn usually require high frequency repetitive stimulations at high intensity [17]. It is also possible that GRP release from primary afferent central terminals in the dorsal horn may require a train of high frequency stimulation, instead of single stimulation. To examine this possibility, superficial dorsal horn neurons were recorded under voltage-clamp with cells held at -60 mV [17] and a train of stimulation containing six pulses was delivered at 25 Hz in dorsal root entry zone (DREZ). The stimulation intensity was increased above the threshold for unmyelinated C fibers (18 V, 0.4 ms) in the presence of CNQX (25 μM), AP-5 (50 μM), Picrotoxin (100 μM) and Strychine (2 μM) [17]. Under this condition, we observed the summation of residual EPSCs (Figure 5A). We tested if RC 3095, a GRPR antagonist, might inhibit the residual EPSCs. RC3095 (3 μM, 10 min) did not significantly block the summated EPSCs (93.6 ± 8.8% from baseline, range from 44.7 to 120.1%, n = 8, Figure 5A&5B). A previous study using the same protocol has demonstrated that the summated EPSCs in most neurons are blocked by substance P NK1 and NK2 receptors antagonists [17]. Taken together, these data suggest that GRP did not directly mediate synaptic transmission at the synapses formed between primary afferent central terminals and superficial dorsal horn neurons.

### The FosGFP positive neurons induced by intradermal histamine received glutamatergic inputs

**Figure 6:**
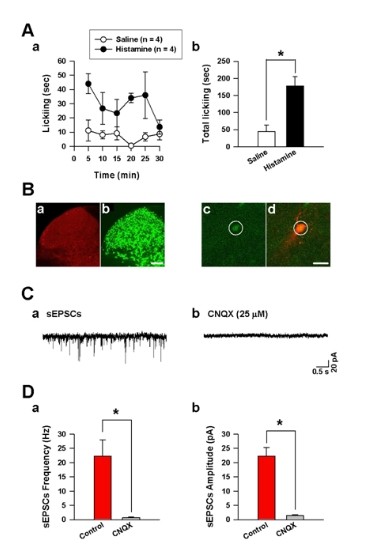
**Identification of the transmitter on FosGFP-expressing neurons in the spinal cord after intradermal histamine injection in FosGFP transgenic mice**. (A) (a) Time course of intradermal histamine (black circles, n = 4) and saline (white circles, n = 4) induced licking behaviors every 5 min for 30 min in FosGFP transgenic mice. (b) The intradermal histamine injection significantly increased total licking behaviors for 30 min compared to the intradermal saline injection. (B) (a) Fos-positive cells were found in the superficial dorsal horn of adult mice 120 min after histamine injection. (b) Dual immunostaining of NeuN in the dorsal horn was also shown. Scale bar, 100 μm. Patch clamp recordings were shown from FosGFP-expressing neurons in the spinal cord. Images showed that one of the FosGFP-expressing neurons (c) in the spinal cord was recorded and labeled by Alexa fluor 594 (d). Yellow color indicated the overlap of GFP and Alexa fluor 594 (d). Scale bar, 20 μm. (C) Spontaneous EPSCs (sEPSCs) recorded on the FosGFP-expressing neurons of the dorsal horn (a) were completely blocked by a bath application of CNQX (25 μM) (b). (D) The summarized data showing that the frequency (a) and amplitude (b) of sEPSCs in histamine induced FosGFP-expressing neurons were totally blocked by CNQX (n = 4).

Recent studies suggest that there are also non-GRP sensitive itching pathways in the spinal cord [3,13]. In order to further investigate whether glutamate could be a major transmitter in itch-activated neurons at the dorsal horn, we used transgenic mice in which the expression of GFP is controlled by the promoter of the *c-fos *gene [18,19]. At the dorsal horn, *c-fos *is known to be activated in sensory neurons after peripheral itch stimulation [20-22]. First, intradermal injections of histamine (500 μg/50 μl) to the hindlimb produced licking behaviors in the mice for 30 min (Figure 6Aa). Intradermal histamine significantly increased the licking behaviors for 30 mini compared with saline injected group (44.5 ± 18.4% in saline group; 151 ± 34.7% in histamine group, unpaired *t *test, *P < 0.05, n = 4 each group, Figure 6Ab). After the observations for thirty minutes, we made lumber part spinal cord slices. Similar to previous studies [20-22], we found that a number of Fos-positive neurons were greatly increased in the spinal cord after intradermal injection of histamine (Figure 6B). The Fos-positive neurons were mainly expressed in the superficial dorsal horn (lamina I & II), especially the lateral part of superficial dorsal horn, with scattered Fos-positive neurons distributed in deep lamina (Figure 6Ba&6Bb). These results are consistent with previous reports in the spinal cord [20-22]. Next, whole-cell patch-clamp recordings were performed from visually identified FosGFP-positive cells ("green" cells) located in the superficial dorsal horn (Figure 6Bc&6Bd). Interestingly, spontaneous EPSCs (sEPSCs) in the FosGFP positive cells of the dorsal horn were completely blocked by CNQX (25 μM) (averaged sEPSCs frequency was 22.3 ± 5.7 Hz in control, 0.7 ± 0.2 Hz in CNQX; averaged sEPSCs amplitude was 22.2 ± 3.1 pA in control, 1.4 ± 0.3 pA in CNQX; unpaired *t *test, * P < 0.05, n = 4, Figure 6C&6D), suggesting that excitatory inputs to itch-activated dorsal horn neurons are glutamatergic.

**Figure 7:**
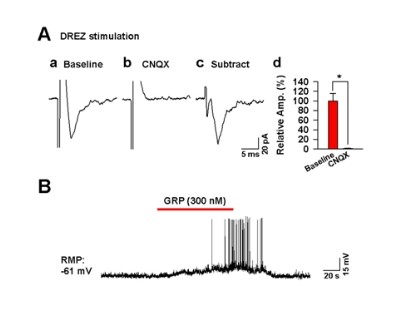
**The characterization of itch-induced FosGFP-expressing neurons in the spinal cord after intradermal histamine injection in FosGFP transgenic mice**. (A) The evoked responses by DREZ stimulation on FosGFP-expressing neurons of the spinal cord (a) were blocked by a bath application of CNQX (25 μM) (b&c). The summarized amplitudes of evoked EPSCs before and after CNQX application was shown in (d) (n = 5). (B) A bath application of GRP (300 nM for 2 min) elicited action potential firing on one FosGFP-expressing neuron in the spinal cord.

To examine if evoked responses between afferent fibers and itch-activated dorsal horn neurons are also mediated by glutamate, we also recorded the evoked EPSCs in FosGFP positive cells by stimulating the DREZ (Figure 7A). Interestingly, the evoked EPSCs were completely blocked by bath applications of CNQX (25 μM) (0.96 ± 0.77% of baseline in CNQX, n = 5, * P < 0.05) (Figure 7A), suggesting that glutamate receptors mediate postsynaptic sensory responses in itch-activated cells. These findings suggest that glutamate is the excitatory transmitter for GRP sensitive or insensitive dorsal horn neurons. Finally, we wanted to determine if histamine induced FosGFP positive cells in the dorsal horn were sensitive to GRP. Interestingly, only one of four cells (25%) were activated by GRP application in FosGFP positive group (n = 4, Figure 7B), suggesting the existence of significant non-GRP sensitive itch-activated dorsal horn neurons.

## Discussion

### The sensitivity of GRP in superficial spinal cord

To our knowledge, the current work represents the first demonstration that glutamate is the major transmitter mediating synaptic transmission from primary afferent fibers onto the GRP-sensitive neurons in the dorsal horn of spinal cord, and itch-activated neurons also received glutamatergic synaptic transmission in the spinal cord. The functional results of GRP positive neurons in the superficial dorsal horn are in consistent with previous anatomic results that GRP positive fibers are restricted to the superficial dorsal horn. Furthermore, GRPRs are mainly expressed in the same area [6,7]. In addition, we found that GRP activated calcium signaling in culture neurons of rat dorsal horn (9%) and GRP elicited action potential firing in superficial dorsal horn neurons (26%) of rats and mice. In some cases, the application of GRP also triggered long-lasting increases of action potentials. These results are found in spinal dorsal horn neurons of both rats and mice. The difference in the percentages of GRP responsive cells in culture vs. *in vitro *spinal cord slices may be due to the different pools of cell recorded. In the case of dorsal horn cultures, all dorsal horn neurons were included, while in spinal cord slices, electrophysiological recordings were only performed from the superficial dorsal horn lamina.

### GRP positive neurons receive monosynaptic C fiber afferents via glutamatergic transmission

It is reported that GRP is anatomically expressed in a subset of small and medium-sized DRG cells from which the GRP positive fibers are restricted to the superficial lamina in the spinal dorsal horn (lamina I and II). Furthermore, GRPRs are mainly expressed in the same area [6,7]. We thus focused monosynaptic evoked responses on the superficial dorsal horn neurons within lamina I and II in this study. In the superficial dorsal horn neurons, the GRP-sensitive neurons mainly received C fiber inputs while few GRP sensitive neurons receive A_δ _fiber. This functional data support previous anatomical observation that GRP is expressed in a subset of small and medium-sized DRG neurons [6]. It is also possible that some of GRP may be released from central origin, such as spinal local neurons or descending projection fibers. We found that the primary afferent mediated responses were blocked by CNQX, suggesting that excitatory transmission is mediated by postsynaptic AMPA and KA receptors in lamina I and II spinal cord. Anatomically, neurons in lamina II compose excitatory or inhibitory interneurons, which have various morphological features and receive primary afferent input predominantly from A_δ _or C fiber [23,24]. Therefore, understanding the anatomical features and electrophysiological property responding to GRP may be one of the key to reveal itch mechanisms in the spinal cord.

### Intradermal histamine induced itch cells in the spinal cord

Histamine is one of the major pruritic molecule to induce itch sensation [3,4,14]. It has been shown that histamine injection could activate the expression of c-Fos in the spinal cord [20]. However, till now, there is no study to investigate synaptic transmission between primary afferent fibers and histamine-activated dorsal horn neurons. In our present study, we used FosGFP transgenic mice to investigate the synaptic transmission on the itch activated FosGFP positive neurons in the dorsal horn by intradermal histamine injection. Histamine induced FosGFP positive neurons were found in the superficial dorsal horn especially at the lateral part, a finding similar to previous reports using immunostaining method. We found that bath applications of CNQX blocked excitatory responses recorded from these dorsal horn neurons, suggesting that again glutamate serves as the major excitatory transmitter. Some of these itch-activated dorsal horn neurons are GRP insensitive, suggesting not all itch-related dorsal horn neurons are GRP sensitive.

### Itch mechanisms in the spinal cord

In the present study, we focused on the superficial dorsal horn neurons receiving monosynaptic inputs from primary afferent fibers. Itch sensation may consist of multiple signaling pathways in the periphery and the dorsal horn. For example, in the spinal dorsal horn, the loss of inhibitory interneurons contributes to elevated itch responses in behavioral animals [8]. Furthermore, two recent studies [10,11] indicate that deletion of VGLUT2 in DRG cells enhanced behavioral itch responses. These findings suggest the possible modulatory roles of glutamatergic transmission in spinal itch circuits. It may be mediated by autosynaptic or heterosynaptic regulation of transmitter release within the spinal cord dorsal horn. Although future studies are clearly needed to determine possible contribution of AMPA and KA receptors to itch, previous studies show that glutamate KA receptors play important roles in the regulation of excitatory as well as inhibitory transmission [25]. Our preliminary data found that mice lacking KA GluR5 subunit showed reduced itch responses (unpublished data). Different signaling pathways are likely involved in different forms of itch responses. Transient receptor potential vanilloid 1 (TRPV1) is known to involve in nociception [1,26,27]. Interestingly, TRPV1-deficient mice showed significant deficits in histamine induced scratching responses. In contrast, neither α-Me-5-HT- nor ET-1-evoked scratching was reduced in the mutant mice [28,29]. In addition, sensory neurons express the G-protein coupled receptor *Mrgpra3 *in the DRG are necessary for itch evoked by chloroquine but not histamine [30].

## Conclusions

In summary, our results provide the first evidence of glutamate mediated excitatory transmission from primary unmyelinated C fibers to GRP sensitive superficial dorsal horn neurons and itch responsive dorsal horn neurons. Glutamate acts as the major excitatory transmitter for itch-related transmission in the spinal cord. Our results also provide the electrophysiological evidence that GRP may contribute to itch sensation by activating subpopulation of spinal dorsal horn neurons that mainly receive unmyelinated C fiber inputs from the periphery. Thus, GRP acts a selective neuromodulator of itch at the spinal level. In addition to itch regulation at the spinal cord, GRP and its receptors have been reported in other spinal cord functions such as male sexual behavior [31], and in supraspinal structures including the amygdala, suprachiasmatic nucleus and the anterior cingulate cortex [32-34]. Future studies are clearly needed to investigate other functions of GRP and the use of GRP/GRPR as potential therapeutic targets for treating itch may have some unwanted CNS side-effects.

## Materials and methods

### Animals

Sprague-Dawley rats (3-8 weeks old) and male C57BL/6 mice (3-8 weeks old) were used in this study. The transgenic FosGFP mice (3-8 weeks old) were obtained from the laboratory of Dr. Alison Barth (Carnegie Mellon University). Experiments were performed under protocols approved by the University of Toronto Animal Care Committee.

### Spinal culture neuron and Ca^2+ ^imaging

Spinal cord dorsal horn neuron cultures in rats (7 to 14 days) were prepared as previously described [12]. Neurons cultured on coverslips were loaded with Ca^2+ ^indicator Fluo-3 (Invitrogen) by incubating cells with 5 μM Fluo-3-AM in a normal bath solution at 35°C for 30 min. The normal bath solution contained (in mM) 150 NaCl, 5 KCl, 2 MgCl_2_, 2 CaCl_2_, 10 glucose, 10 HEPES, pH 7.3, 320 mOsm was used. After dye loading, a coverslip was mounted on a 0.5 ml perfusion chamber and the chamber was then placed on the stage of an inverted Olympus IX70 microscope (Lake Success, NY). Fluo-3 was excited at 450 nm with a mercury lamp and fluorescence emission was collected at 550 nm. Fluo-3 fluorescence in the cells was detected with a peltier-cooled charge-coupled device camera (PentaMAX-III System, Roper Scientific, Trenton, NJ) under a 10× objective. Images were acquired at one frame per second, 200 ms exposure time per frame. Neurons were tested for their sensitivity to GRP by applying 300 nM GRP solution for 10 seconds through a glass tube (~500 μm ID) positioned 1.0 mm away from cells at room temperature. Relative fluorescence intensity (ΔF/F_0_) was used to represent GRP responses and neurons with ΔF/F_0 _values of ≥ 0.15 (i.e., equal or above 15% baseline fluorescence intensity) were considered as responsive cells [12].

### Whole-cell patch-clamp recordings

Transverse slices (400-600 μm) of the lumbar spinal cord attached with L4 or L5 dorsal roots (8-14 mm) were prepared as previous described [35-37]. The oxygenated (95% O_2 _and 5% CO_2_) artificial cerebrospinal fluid containing (in mM) 124 NaCl, 2.5 KCl, 2 CaCl_2_, 1 MgSO_4_, 25 NaHCO_3_, 1 NaH_2_PO_4_, and 10 glucose at room temperature was used. Neurons in lamina I/II of the spinal dorsal horn were recorded with an Axon 200B amplifier (Molecular device, Union city, CA). Recording electrodes (3-5 MΩ) contained an internal solution composed of (in mM): 124 K-gluconate, 5 NaCl, 1 MgCl_2_, 0.2 EGTA, 10 HEPES, 2 Mg-ATP, 0.3 Na_3_GTP, pH 7.2; 280-300 mOsm. Action potentials were recorded in current clamp mode (I = 0). The dorsal root evoked excitatory postsynaptic currents (EPSCs) were induced by repetitive stimulations at 0.02 Hz via a suction electrode, and neurons were voltage-clamped at -60 mV in the presence of AP-5 (50 μM). Picrotoxin (100 μM) and Strychine (2 μM) were also present to block γ-aminobutyric acid (A) (GABA_A_) and glycine receptors, respectively. A_δ _or C fiber-evoked EPSCs were distinguished on the basis of the conduction velocity of afferent fibers (A_δ_, 2-13 m/s; C, < 0.8 m/s), and monosynaptic responses were identified by measuring no failure by repetitive dorsal root stimulations (20 Hz, 20 times for A_δ _and 2 Hz, 20 times for C fiber) [15]. Focal EPSCs were evoked at a frequency of 0.03 Hz delivered by bipolar tungsten stimulating electrode place at the dorsal root entry zone (DREZ) of the spinal cord [16,38]. In order to observe neuropeptides-mediated EPSCs, a train of stimulation containing six pulses delivered at 25 Hz was used by bipolar tungsten stimulating electrode placed at DREZ of the spinal cord, and neurons were voltage-clamped at -60 mV in the presence of CNQX (25 μM), AP-5 (50 μM), Picrotoxin (100 μM) and Strychine (2 μM). The initial access resistance was 15-30 MΩ, and it was monitored throughout the experiment. Data were discarded if the access resistance changed > 15% during experiment. Data were filtered at 1 kHz, and digitized at 10 kHz.

### Histamine injection and behaviors

FosGFP mice were shaved at the hindlimb where intradermal injection of histamine (500 μg/50 μl). Hindlimb licking behavior directed towards the shaved area at the hindlimb was observed for 30 min at 5-min intervals [6,7]. After the observations, the spinal cord slices at lumber L4 and L5 were made, and intradermal histamine induced fos positive neurons were recorded from the superficial dorsal horn.

### Data analysis

Statistical comparisons were made using the unpaired *t*-test. All data were presented as the Mean ± S.E.M. In all cases, * *P *< 0.05 was considered statistically significant.

## List of Abbreviations

AMPA: 2-amino-3-(5-methyl-3-oxo-1,2-oxazol-4-yl) propanoic acid; APs: action potentials; CNQX: 6-cyano-7-nitro-quinoxaline-2,3-dine; DREZ: dorsal root entry zone; DRG: dorsal root ganglion; EPSCs: excitatory postsynaptic currents; GFP: green fluorescent protein; GRP: gastrin releasing peptide; GRPRs: gastrin releasing peptide receptors; KA: kainite; TRPV1: transient receptor potential vanilloid 1; VGLUT2: vesicular glutamate transporter subtype 2.

## Competing interests

The authors declare that they have no competing interests.

## Authors' contributions

KK, TC and XL performed electrophysiology, confocal experiments and drafted the manuscript. KK and GD participated in behavioral test. JL carried out calcium imaging experiments. MZ and JGG designed and finished the final draft of the manuscript. All authors read and approved the final manuscript.
